# Discovery of a Novel *er1* Allele Conferring Powdery Mildew Resistance in Chinese Pea (*Pisum sativum* L.) Landraces

**DOI:** 10.1371/journal.pone.0147624

**Published:** 2016-01-25

**Authors:** Suli Sun, Haining Fu, Zhongyi Wang, Canxing Duan, Xuxiao Zong, Zhendong Zhu

**Affiliations:** National Key Facility for Crop Gene Resources and Genetic Improvement, Institute of Crop Science, Chinese Academy of Agricultural Sciences, Beijing, China; McGill University, CANADA

## Abstract

Pea powdery mildew, caused by *Erysiphe pisi* D.C., is an important disease worldwide. Deployment of resistant varieties is the main way to control this disease. This study aimed to screen Chinese pea (*Pisum sativum* L.) landraces resistant to *E*. *pisi*, and to characterize the resistance gene(s) at the *er1* locus in the resistant landraces, and to develop functional marker(s) specific to the novel *er1* allele. The 322 landraces showed different resistance levels. Among them, 12 (3.73%), 4 (1.24%) and 17 (5.28%) landraces showed immunity, high resistance and resistance to *E*. *pisi*, respectively. The other landraces appeared susceptible or highly susceptible to *E*. *pisi*. Most of the immune and highly resistant landraces were collected from Yunnan province. To characterize the resistance gene at the *er1* locus, cDNA sequences of *PsMLO1* gene were determined in 12 immune and four highly resistant accessions. The cDNAs of *PsMLO1* from the immune landrace G0005576 produced three distinct transcripts, characterized by a 129-bp deletion, and 155-bp and 220-bp insertions, which were consistent with those of *er1*-2 allele. The *PsMLO1* cDNAs in the other 15 resistant landraces produced identical transcripts, which had a new point mutation (T→C) at position 1121 of *PsMLO1*, indicating a novel *er1* allele, designated as *er1*-6. This mutation caused a leucine to proline change in the amino acid sequence. Subsequently, the resistance allele *er1*-6 in landrace G0001778 was confirmed by resistance inheritance analysis and genetic mapping on the region of the *er1* locus using populations derived from G0001778 × Bawan 6. Finally, a functional marker specific to *er1*-6, SNP1121, was developed using the high-resolution melting technique, which could be used in pea breeding via marker-assisted selection. The results described here provide valuable genetic information for Chinese pea landraces and a powerful tool for pea breeders.

## Introduction

Pea (*Pisum sativum* L.) is an important legume crop that is distributed worldwide in more than 90 countries [1]. In China, peas have been cultivated for more than 2,000 years [2]. To date, China is the world’s largest and third largest producer of green peas and dry peas, respectively [1]. Pea powdery mildew, caused by *Erysiphe pisi* D.C., is a major pea disease worldwide, which results in heavy losses in yield and quality [3, 4]. The powdery mildew caused by *E*. *pisi* can induce yield losses of 25–50% in pea production [5–7]. Severe infection by *E*. *pisi* can result in yield loss up to 80% in susceptible cultivars in temperate and subtropical regions with warm, dry days and cool nights [4, 8]. The deployment of genetically resistant varieties has been considered an efficient, economic and environmentally friendly way to control this disease [8, 9].

Until a few years ago, *E*. *pisi* was thought to be the only pathogen causing powdery mildew in peas. Thus, great effort and attention has been paid to breeding peas resistant to *E*. *pisi* in pea primary producing countries, although the other two *Erysiphe* species, *E*. *trifolii* and *E*. *baeumleri*, also attack peas and show the same disease symptoms as *E*. *pisi* in India, the USA and the Czech Republic [10, 11]. Many resistant pea germplasms have been identified since Harland initiated research on pea powdery mildew resistance in 1948 [12]. In a few of them, their resistance gene(s) to *E*. *pisi* have been revealed by genetic analysis [13]. Two single recessive genes (*er1* and *er2*) and one dominant gene (*Er3*) have been identified for powdery mildew resistance in pea germplasms to date [12, 14, 15]. Moreover, pea molecular markers linked to the three resistance genes (*er1*, *er2* and *Er3*) have been developed by several research groups [16–24]. The single recessive genes *er1* and *er2* have been mapped to pea linkage groups (LGs) VI and III, respectively [17, 21, 25], whereas *Er3* was mapped between the sequenced characterized amplified region (SCAR) marker Scw4_637_ and random amplified polymorphic DNA (RAPD) marker OPAG05_1240 located on an uncertain pea LG [26].

Resistance gene *er2* is only harbored by a few resistant pea germplasms, including SVP951, SVP952 and JI 2480 [27]. The expression of resistance gene *er2* is strongly influenced by temperature and leaf age. The complete resistance conferred by *er2* is expressed only at high temperature (25°C) or in mature leaves, and is based mainly on post-penetration cell death, mediated by a hypersensitive response [28]. Moreover, although *er2* confers completely effective powdery mildew resistance in some locations, it is ineffective in others [14, 27–29]. *Er3* is a newly identified dominant gene from a wild relative of pea (*P*. *fulvum*), which has been successfully introduced into cultivated pea (*P*. *sativum*) recently [7, 15]. In *Er3* plants, most of the *E*. *pisi* conidia are able to penetrate the pea epidermal cells and form secondary hyphae, but growth of these established colonies is prevented by a strong hypersensitive response [15, 30].

The gene *er1* is widespread in resistant pea germplasms and is considered as a stable, durable and broadly effective resistance gene that inhibits *E*. *pisi* invasion of pea epidermal cells [28]. Several genetic analyses of resistance to *E*. *pisi* indicated that the vast majority of resistant pea germplasms contained resistance gene *er1*, which has been used successfully for decades in pea breeding programs [27], because *er1* provides complete immunity or high levels of powdery mildew resistance [27, 28, 29, 31–33]. Recent studies indicated that the resistance conferred by *er1* in peas is similar to that in some monocot (barley) and dicot (*Arabidopsis thaliana*, tomato) plants [13, 34]. The *er1*-resistant phenotype is caused by loss-of-function mutations of a pea MLO (Mildew Resistance Locus O) homolog, named *PsMLO1*, a powdery mildew susceptibility gene [13, 34]. To date, five *er1* alleles (*er1*-1, *er1*-2, *er1*-3, *er1*-4 and *er1*-5) have been discovered in pea resistant germplasms, which were produced by natural or artificial mutagenesis. Each of the five *er1* alleles corresponds to a different *PsMLO1* mutation, according to the mutation site and pattern of *PsMLO1* [13, 34, 35]. Among the reported five *er1* alleles, *er1*-1 and *er1*-2 are used commonly in pea breeding programs [13, 34].

In China, powdery mildew caused by *E*. *pisi* seriously affects the yield and quality of peas [36]. The incidence of pea powdery mildew has reached 100% in some regions of China [37]. Currently, *E*. *pisi* is thought to be the only causative agent of pea powdery mildew in China [36–40], although the other two species, *E*. *trifolii* and *E*. *baeumleri*, were identified on peas in other regions [10, 11]. Ondřej et al. [10] and Attanayake et al. [11], respectively, proved that *E*. *baeumleri* and *E*. *trifolii* were able to overcome *er1* gene-induced resistance to *E*. *pisi*. Recently, Fondevilla et al. [41] further confirmed that *E*. *trifolii* could also overcome resistance gene *Er3*, but not *er2*.

In China, screenings of pea germplasms’ resistance to powdery mildew (*E*. *pisi*) have been carried out in fields and greenhouses [37–40]. The results revealed that several Chinese pea germplasms were immune and highly resistant to *E*. *pisi* isolates EPBJ (NCBI, accession number KR912079) and EPYN (NCBI, accession number KR957355). Recently, Wang et al. [39] reported Chinese pea landraces showing resistance to *E*. *pisi*, especially those collected from Yunnan province. To date, there has been no detailed research on the resistance of Chinese pea landraces against *E*. *pisi*. The genetic basis of resistance to powdery mildew and the related resistance gene(s) harbored in Chinese pea landraces are unknown. Therefore, this study was designed to screen Chinese pea landraces for resistance to *E*. *pisi*, and to identify and characterize the resistance alleles at the *er1* locus by determining the cDNA sequence of the *PsMLO1* gene in resistant pea landraces. We also aimed to genetically map the discovered novel *er1* allele and develop functional marker(s) specific to the novel *er1* allele for better marker-assisted selection in pea breeding programs.

## Materials and Methods

### 2.1. Phenotypic assessment of Chinese pea landraces

#### 2.1.1 Plant material and inoculation

Three hundred and twenty-two accessions of pea landraces collected from 18 Provinces or Autonomous Regions of China (including Gansu, Guizhou, Hebei, Henan, Hubei, Jiangsu, Jiangxi, Inner Mongolia, Ningxia, Qinghai, Shandong, Shanxi, Shaanxi, Sichuan, Tibet, Xinjiang, Yunnan and Chongqing) and preserved in China National Genebank were selected to evaluate their resistance to powdery mildew (*E*. *pisi*) (Table 1). The pea cultivar(cv.) Bawan 6 harboring the susceptible gene *Er1* was used as the susceptible control [42]. Pea line X9002 developed from Gansu province and harboring *er1*-2, and pea cultivar YI (JI1591) originating from Guangdong province and harboring *er1*-4, were used as resistant controls [13, 42].

**Table 1:** Geographical distribution of 322 Chinese pea landraces and their phenotypic reaction to *E*. *pisi* isolate EPYN.

Distribution	Total No. of landrace	Total No. of resistance	Phenotypic reaction to *E*. *pisi*				
			Resistant (score)			Susceptible (score)	
			I(0)	HR(1)	R(2)	S(3)	HS(4)
Gansu	35	0	0	0	0	17	18
Guizhou	27	0	0	0	0	1	26
Hebei	8	2	0	1	1	2	4
Henan	4	0	0	0	0	0	4
Hubei	2	0	0	0	0	0	2
Jiangsu	2	0	0	0	0	0	2
Jiangxi	2	0	0	0	0	0	2
Inner Mongolia	23	2	0	0	2	18	3
Ningxia	25	6	0	0	6	7	12
Qinghai	1	0	0	0	0	0	1
Shandong	1	0	0	0	0	0	1
Shanxi	3	1	0	0	1	1	1
Shaanxi	46	3	0	0	3	14	29
Sichuan	22	0	0	0	0	11	11
Xizang	45	0	0	0	0	21	24
Xinjiang	12	0	0	0	0	2	10
Yunnan	46	18	11	3	4	11	17
Chongqing	18	1	1	0	0	16	1
Total No. of Landrace	322	33	12	4	17	121	168

"I, HR, R, S, HS" indicate resistance levels; I: immunity; HR: high resistance; R: resistance; S: susceptible; HS: high susceptibility.

The seeds of 322 accessions and controls were planted in 15-cm-diameter paper pots (six seeds/pot) filled with a mixture of vermiculite and peat moss (1:1). Thirty seeds of each pea landrace and controls were planted with five replications. The planted pots were placed in a greenhouse at 18–26°C. Fourteen days after planting, the plants at the fourth or fifth leaf stage were inoculated with *E*. *pisi* isolate EPYN (NCBI, accession number KR957355) collected from a field in Yunnan [39, 40], using conidia and by shaking heavily infected plants of pea cv. Longwan 1. Ten days after inoculation, disease severity was scored using a scale of 0–4, according to the infected foliage area, macroscopic and microscopic observation of mycelia growth and sporulation [29, 39, 40, 43] (S1 Fig). 0 = No colony or mycelium growth on inoculated leave; 1 = few colony with a film of mycelium growth and mycelium with very little sporulation; 2 = a few countable colonies with evident mycelium growth and mycelium with slight sporulation; 3 = uncountable colonies with abundant growth mycelium and mycelium with moderate to heavy sporulation; 4 = infected leaves fully covered by abundant growth mycelium with heavy to very heavy sporulation. Plants with scores of 0, 1, and 2 were classified as immune, highly resistant and resistant, respectively, and those with scores of 3 and 4 were classified as susceptible and highly susceptible, respectively. The test was repeated for the pea landraces identified as resistant to *E*. *pisi*.

### 2.2 cDNA sequence analysis of *PsMLO1* gene at *er1* locus

The pea landraces identified as immune (four accessions) and highly resistant (12 accessions) to *E*. *pisi* were analyzed to identify their resistance allele at *er1* locus by determining the cDNA sequence of the *PsMLO1* gene.

Total RNA was extracted from the young leaves of the 16 resistant landraces (Table 2) and three controls (G0005658, G0006273 and P000I391630), using an RNAprep Pure Plant kit (Tiangen Biotech, Beijing, Co., Ltd), according to the manufacturer’s instructions. First-strand cDNAs were synthesized using a BioRT Two Step RT-PCR Kit (Hangzhou Bioer Technology Co., Ltd) including an oligo (dT) primer. The PCR reactions for the full-length cDNA of the target gene, *PsMLO1*, used the *PsMLO1*-specific primers PsMLO1F/PsMLO1R (5′-AAAATGGCTGAAGAGGGAGTT-3′/5′-TCCACAAATCAAGCTGCTACC-3′) [34]. The amplification reaction was carried out under the following conditions: 5 min at 95°C; 35 cycles of 30 s at 94°C, 45 s at 58°C and 1 min at 72°C, with a final extension of 10 min at 72°C. The amplicons were purified using a PCR Purification Kit (Qiagen) and ligated into the pEasy_T5 vector (TransGen Biotech, Beijing, Co., Ltd). Sequencing reactions were performed at the Beijing Genomics Institute (BGI), and 10 clones were sequenced for each landrace. The resulting cDNA sequences of the *PsMLO1* gene from the 16 resistant landraces and controls were analyzed using ClustalX2 and compared with the reference cDNAs of *PsMLO1* in the wild-type pea cv. Sprinter (susceptible to *E*. *pisi*) (NCBI, accession number FJ463618.1) [13].

**Table 2:** Resistance reactions to *E*. *pisi* isolate EPYN of Chinese pea landraces and identified resistance alleles of *er1*.

Accession No.	Landrace/Cultivar name	Province of Origin	Reaction to *E*. *pisi*	*er1* allele	Reference
G0005658	Bawan 6	Hebei	S(4)	*Er1*	Wang et al. ^[^^42^^]^
G0006273	X9002	Gansu	I(0)	*er1-2*	Wang et al. ^[^^42^^]^
PI391630	YI	Guangdong	I(0)	*er1-4*	Humphry et al. ^[^^13^^]^
G0001752	Wandou	Yunnan	I(0)	*er1-6*	this study
G0001763	Baiwandou	Yunnan	I(0)	*er1-6*	this study
G0001764	Dabaiwandou	Yunnan	I(0)	*er1-6*	this study
G0001767	Fanwandou	Yunnan	I(0)	*er1-6*	this study
G0001768	Wandou	Yunnan	I(0)	*er1-6*	this study
G0001777	Semanuo	Yunnan	I(0)	*er1-6*	this study
G0001778	Dabaiwandou	Yunnan	I(0)	*er1-6*	this study
G0001780	Dabaiwandou	Yunnan	I(0)	*er1-6*	this study
G0003824	Wandou	Yunnan	I(0)	*er1-6*	this study
G0003825	Dajiewandou	Yunnan	I(0)	*er1-6*	this study
G0003826	Wandou	Yunnan	I(0)	*er1-6*	this study
G0003831	Dawandou	Yunnan	R(1)	*er1-6*	this study
G0003834	Dabaiwandou	Yunnan	R(1)	*er1-6*	this study
G0003836	Xinjiewandou	Yunnan	R(1)	*er1-6*	this study
G0003694	Baiwandou	Hebei	R(1)	*er1-6*	this study
G0005576	Wandou	Chongqing	I(0)	*er1-2*	this study

"I, R, S" indicate resistance levels; "0, 1, 4" indicate disease scores.

### 2.3 Genetic analysis of G0001778 carrying novel allele *er1-6*

#### 2.3.1 Parents and generated genetic populations

The pea landrace G0001778 (immune to *E*. *pisi* isolate EPYN) and susceptible control cv. Bawan 6 were used as parents and were crossed to generate genetic populations. The derived F_1_, F_2_ and F_2:3_ populations from G0001778 × Bawan 6 were used for phenotypic evaluation and genetic analysis for resistance to *E*. *pisi* in G0001778.

#### 2.3.2 Phenotypic evaluation

Planting of parents, and the F_1_ and F_2_ populations derived from G0001778 × Bawan 6, was performed in a propagation greenhouse to generate seeds of F_2_ and F_2:3_ families. Inoculation of plants at the fourth or fifth leaf stage was performed using the detached leaf method under controlled conditions. The third or fourth leaves, along with petiole from the bottom, were collected from each F_1_ and F_2_ seedling. The detached leaves were inserted into 2% water agar containing 50 mg/L benzimidazole in 9-cm-diameter Petri dishes [44], and were then inoculated with conidia of isolate EPYN using the same method as the inoculation of the landrace plants [42]; i.e., by shaking plants of cv. Longwan 1 with a mass of conidia. After inoculation, the treated plates were sealed tightly with Parafilm, and placed in a growth chamber at 20°C with a 14-h photoperiod. The detached leaves of both parents, G0001778 and Bawan 6, were treated in the same way with isolate EPYN as controls. The disease severity was determined using a scale of 0–4 as stated above (S1 Fig), 10 days after inoculation. Plants with scores of 0–2 and 3–4 were classified as resistant and susceptible, respectively [29, 43]. The plants considered resistant to *E*. *pisi* were subjected to a repeated test.

Twenty-four seeds selected randomly from each F_2:3_ family were planted in paper pots. Planting design and incubation of plants were performed as in section 2.1.1, together with the parents as resistant and susceptible controls. Disease severity was rated using the 0–4 scale as for the F_2_ population 10 days after inoculation. The families classified as either homozygous resistance, or those segregated for resistance and susceptibility to *E*. *pisi*, were subjected to repeated tests.

A chi-squared (χ^2^) test was used to determine the goodness-of-fit to Mendelian segregation patterns of the phenotypes of the F_2_ and F_2:3_ populations from G0001778 × Bawan 6.

#### 2.3.3 Genetic mapping of resistance allele *er*1-6

Genomic DNA was isolated from healthy young leaves collected from seedlings of parents, G0001778 and Bawan 6, and the derived F_2_ population using the cetyl trimethylammonium bromide (CTAB) extraction method [45]. The DNA concentration was calculated on an ND-1000 Nano-Drop (NanoDrop Technologies Inc., Wilmington, DE, USA). The DNA solution was diluted to a working concentration of 50 ng/μL with Tris–EDTA buffer (pH 8.0) and stored at −20°C until use.

The molecular markers distributed on pea LG VI and known to be linked to the *er1* locus, including five simple sequence repeat (SSR) markers (PSMPSAD51, PSMPSA5, PSMPSAD60 i.e. AD60, PSMPSAA374e and PSMPSAA369) [18, 46, 47], and a gene marker Cytosine-5 (DNA-methyltransferase, c5DNAmet) [42, 48], were selected and used to screen for polymorphisms between the contrasting parents. The obtained polymorphic markers between the parents were further used to determine the genotypes of each F_2_ plant for genetic linkage analysis. The linkage analysis could reveal the marker(s) linked to resistance gene and the location of the resistance gene.

PCR amplification of the molecular markers was conducted in a total volume of 20 μl, containing 50 ng of genomic DNA, 2.5 μl 10×PCR reaction buffer (20 mM MgCl_2_), 0.2 mM each dNTP, 1.5 U of *Taq* DNA polymerase and 0.2 μM of primer mixture. PCR reactions were carried out at 94°C for 5 min, followed by 35 cycles of 94°C for 30 s, 49–60°C (depending on the primer-specific annealing temperature) for 30 s and 72°C for 1 min, with a final extension at 72°C for 10 min, using a thermal cycler (Biometra, Goettingen, Germany). The amplified PCR products were separated on a 6% polyacrylamide gel.

The segregation data of polymorphic markers in the F_2_ population were evaluated for goodness-of-fit to Mendelian segregation ratios by a chi-squared test. Genetic linkage analyses were performed using MAPMAKER/EXP version 3.0b [49] and the genetic distance was computed using the Kosambi mapping function [50]. A logarithm of odds (LOD) score of 3.0 and a maximum distance of 50 cM were used as the threshold to determine the linkage groups. The genetic linkage map was drawn with molecular markers linked to the resistance gene using MapDraw [51].

### 2.4 Development of a functional marker specific for *er1-6*

**Fig 1:**
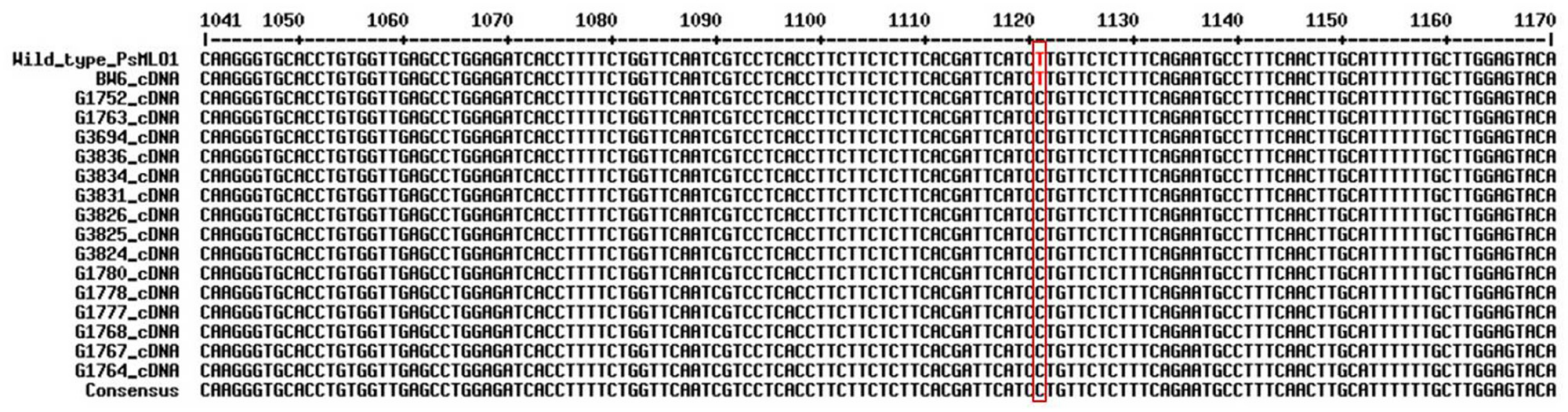
Comparisons of the cDNA sequence of the *PsMLO1* gene from the wild-type pea cultivar Sprinter (Wild_type_PsMLO1) and Bawan 6 (BW6_PsMLO1_cDNA), which are susceptible to *E*. *pisi*, and the 16 resistant pea landraces. Mutated sites in the *PsMLO1* cDNA sequence are boxed in red.

Based on the cDNA sequence difference of the *PsMLO1* gene between the contrasting parents, G0001778 and Bawan 6, five primer pairs flanking the mutation site (T→C) were designed to develop a SNP functional marker specific to the novel allele *er1*-6, using software Primer Premier 5.0 (Fig 1 and S1 Table). The SNP marker was analyzed by a high-resolution melting (HRM) assay [52].

DNAs of the parents, G0001778 and Bawan 6, and their derived F_2_ population comprising 71 individuals, were used as templates of PCR amplification. Later, to verify the validity of the developed SNP marker, PCR amplifications were also performed on the five susceptible pea germplasms (Bawan 6, Longwan 1, G0001747, G0003839, G0003840) carrying susceptible *Er1* gene, and resistant germplasms, Tara [40], X9002 [42] and G0005576, and YI [13] respectively carrying *er1*-1, *er1*-2 and *er1*-4 alleles, and the identified resistant pea landraces (G0001752, G0001763, G0001764, G0001767, G0001768, G0001778, G0001780, G0003824) carrying the *er1*-6 allele.

For the HRM assay, the PCR reactions and amplifications by the five primers were conducted in a 96-well plate and performed in a total volume of 10 μl, containing 25 ng of genomic DNA, 1 μl 10×PCR reaction buffer (25 mM MgCl_2_), and 0.5 μl (5 mM) primer mixture, 5 μl 10×HRM Fast Master Mix (HRM Fast PCR Kit, Kapa Biosystems, USA). PCR reactions were conducted at 95°C for 5 min, followed by 50 cycles of 95°C for 10 s, 55°C–60°C (depending on the primer-specific annealing temperature) for 30 s and 72°C for 10 s, with a final extension at 72°C for 3 min, using a thermal cycler. For HRM analysis, the amplified PCR products were heated to 95°C and melted at a ramp of 65°C–95°C in a LightCycle 480 (Roche Diagnostics, Mannheim, Germany). The fluorescence data were subsequently visualized and results of the melting curve were analyzed using Roche480 software.

### 2.5 Ethics Statement

No specific permissions were required for domestic research of the collections of pea landraces accessions in China. All the studies did not involve endangered or protected species.

## Results

### 3.1 Phenotypic assessment of pea landraces

In this study, 322 pea landraces collected from 18 Provinces or Autonomous Regions of China were evaluated for powdery mildew resistance to *E*. *pisi*. Ten days after inoculation, the plants of susceptible control Bawan 6 showed severe infection by *E*. *pisi* isolate EPYN under greenhouse conditions. All plants of Bawan 6 were covered by masses of conidia and mycelium of *E*. *pisi*, corresponding to a disease severity of 4 (S1 Fig). By contrast, the resistant controls X9002 and YI appeared to be immune to *E*. *pisi* isolate EPYN, with no symptoms observed. These results are consistent with previous observations [13, 39, 40, 42].

The phenotypic reactions of the 322 pea landraces showed various levels of resistance to *E*. *pisi*, involving immunity, high resistance, resistance, susceptibility, and high susceptibility, corresponding to disease severity 0–4 scales (S1 Fig and Table 1). Among the 322 accessions, 12 (3.72%), four (1.24%) and 17 (5.28%) accessions showed immunity, high resistance and resistance to *E*. *pisi* isolate EPYN (Table 1). One hundred and twenty-one (37.6%) and 168 (52.2%) accessions showed susceptibility and high susceptibility to *E*. *pisi* isolate EPYN (Table 1).

The 33 (10.25%) accessions that showed resistance to isolate EPYN with 0–2 scores were collected from seven provinces of China (Table 1). No resistance resource was found in landraces collected from the other 11 provinces (Table 1). Eighteen out of 33 resistant landraces were derived from Yunnan province, which has a rich diversity of pea germplasms [53]. The 18 accessions represented 39.1% of the 46 tested accessions from Yunnan, which indicated that the highest frequency of resistant resources existed in Yunnan. Moreover, 11 out of 18 resistant pea landraces from Yunnan showed immunity to *E*. *pisi* (Tables 1 and 2). These 11 accessions represented 91.7% of the 12 immune pea landraces (Table 2). The remaining immune landrace, G0005576, was collected from Chongqing (Table 2). Three out of four highly resistant accessions were also collected from Yunnan (Table 2). The remaining landrace, G0003694, was collected from Hebei. The other 17 accessions resistant to *E*. *pisi* with a score of 2 were collected from five provinces (Table 1).

### 3.2 cDNA sequence analysis of *PsMLO1* gene at *er1* locus

RNA was extracted from the 16 pea landraces with immunity and high resistance to powdery mildew and the cDNA sequences of their *PsMLO1* genes at *er1* locus were determined. The *PsMLO1* cDNA sequence of the susceptible control, Bawan 6, was 100% identical to that from the wild-type *PsMLO1* gene. Resistant control pea YI, harboring the *er1*-4 allele, had a single base pair (A) deletion at position 91 of the *PsMLO1* cDNA sequence. Resistant pea line X9002, harboring the *er1*-2 allele, produced three distinct transcripts. The results of the controls agreed with previous studies [13, 42], supporting the accuracy of the sequencing of the target *PsMLO1* cDNA in the applied 16 resistant landraces.

In one of the 16 accessions, G0005576, the cDNA sequences of *PsMLO1* appeared as three distinct transcripts, characterized by a 129-bp deletion, and 155-bp and 220-bp insertions in the wild-type *PsMLO1* of pea cv. Sprinter (S2 Fig), which is identical to the resistant control X9002 [42]. This indicated that the resistance in G0005576 was conferred by *er1* allele, *er1*-2. The cDNA sequences of *PsMLO1* gene in the other 15 resistant landraces showed an identical mutation pattern, comprising a single base pair variation at position 1121 (T→C) of the wild-type *PsMLO1* gene (Fig 1), which was a different mutational event from all known *er1* alleles [13, 34]. This point mutation caused leucine to be replaced by proline, which probably resulted in a functional change of the *PsMLO1* protein. This mutation occurred in these Chinese landraces indicated a novel *er1* allele, designated as *er1*-6, according to previous nomenclature of *er1* alleles [13, 34].

Based on 10 clone sequences, five accessions also had frameshift mutations with small fragments in one or two clone sequences. The cDNA sequences of the *PsMLO1* gene from G0001763 and G0003831 showed a 5-bp (GTTAG) deletion at position 700 of the wild-type *PsMLO1* cDNA in one and two clones, respectively. For G0003831, one cDNA sequence of the *PsMLO1* gene had an 11-bp (GTAGGAATAAG) deletion at position 858 of *PsMLO1*. Another cDNA sequence of *PsMLO1* from G0003831 showed an 88-bp (TAAGTTGATTTCTGAAACAAAAGAGCACCTAATGATATTAACAATTATATATTAAACTAACTATGAAGTGACAATTTTAATTTGCAGG) insertion at position 1241 of *PsMLO1*. One cDNA sequence of *PsMLO1* from G0001764 and G0003694 showed a 16-bp (CTCATCTTCCTCCAGG) deletion at position 776 of *PsMLO1*. One cDNA sequence of the *PsMLO1* gene in G0001778 showed a 13-bp (GTAATCTTATTAG) deletion at position 961 of *PsMLO1*. These deletions or insertions of small fragments in *PsMLO1* cDNAs were assumed to be aberrant splicing events during the process of transcription.

### 3.3 Analysis of genetic resistance in G0001778

The resistant and susceptible parents, G0001778 and Bawan 6, showed the contrasting phenotypes with disease severities of 0 and 4, respectively, under greenhouse conditions.

The segregation patterns of resistance to powdery mildew in the F_1_, F_2_ and F_2:3_ populations derived from G0001778 × Bawan 6 are shown in Table 3. Two F_1_ plants were susceptible to *E*. *pisi* (Table 3). One F_1_ plant produced 71 progeny in the F_2_ and F_2:3_ populations by self-crossing. Among these 71 F_2_ plants, 21 were resistant (R) and 50 were susceptible (S). Thus, the segregation of susceptibility and resistance in F_2_ population fitted a 3:1 ratio (χ^2^ = 0.20; *P* = 0.66), indicating monogenic recessive inheritance. Moreover, the segregation ratio in the F_2:3_ population showed a good fit to a 1 (homozygous susceptible): 2 (segregating): 1 (homozygous resistant) ratio (χ^2^ = 0.70, *P* = 0.70) (Table 3), confirming that resistance in landrace G0001778 was controlled by a single recessive gene.

**Table 3:** Genetic analysis of pea landrace G0001778 carrying the *er1*-6 allele for powdery mildew resistance (*E*. *pisi*).

Parents and the cross	Generation	Amount	No. of plant or families			Expected ratio and goodness of fit		
			R	Rs	S	R:Rs:S	χ^2^	P
G0001778	P1	30	30	-	-	-		
Bawan 6	P2	30	-	-	30	-		
	F_1_	2	-	-	2	-		
G0001778×Bawan 6	F_2_	71	21	-	50	1:3	0.20	0.66
	F_2:3_	71	21	35	15	1:2:1	0.70	0.70

R: resistance; S: susceptible; Rs: segregated.

**Fig 2:**
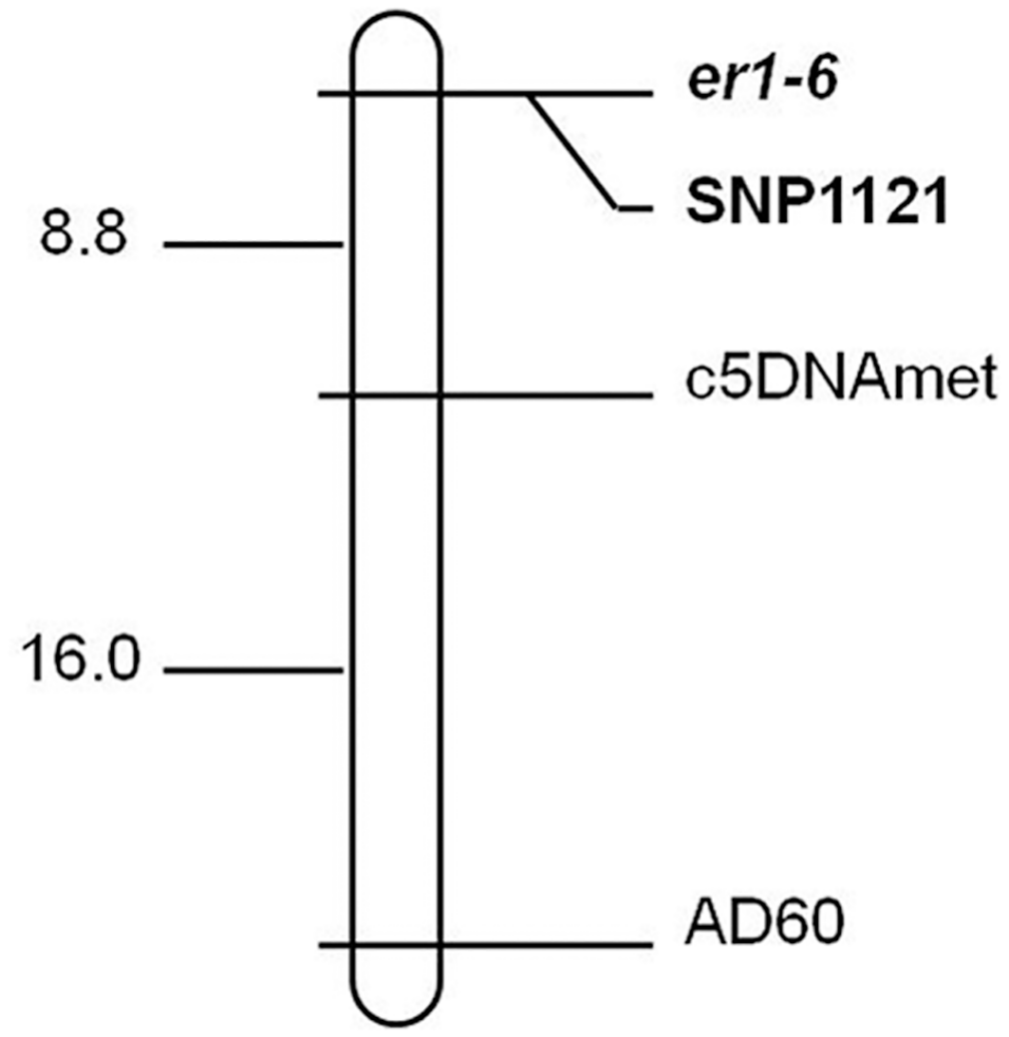
Genetic linkage map of the F_2_ population derived from G0001778 × Bawan 6 with the linked markers AD60 and c5DNAmet, showing the position of the resistance gene for powdery mildew. Map distances and loci order were determined using MAPMAKER 3.0 (Lander et al. 1993). Estimated genetic distances between loci are shown on the left in centiMorgans (cM).

Among the tested markers known to be linked to the *er1* locus, we found that only the SSR marker AD60 and the gene marker c5DNAmet on pea LG VI were polymorphic between the two parents, which were likely to be linked to the resistance gene. Thus, markers AD60 and c5DNAmet were used to determine the genotypes of the whole F_2_ population. Both polymorphic markers revealed that their segregation pattern fitted the 1:2:1 (susceptible homozygotes: heterozygotes: resistant homozygotes) ratios in the F_2_ population, which confirmed that AD60 and c5DNAmet were co-dominant markers. Further genetic linkage analysis confirmed that AD60 and c5DNAmet were linked to the resistance gene (Fig 2). Our results were consistent with previous studies that showed that markers AD60 and c5DNAmet were linked to the *er1* locus [18, 42], which also proved that the resistance gene in G0001778 was located on pea LG VI harboring the *er1* locus.

Linkage analysis of segregation data of a mapping population derived from G0001778 × Bawan 6 was performed to construct genetic linkage maps using MapDraw. Two linked markers AD60 and c5DNAmet were located on the same side of the resistance gene at genetic distances of 8.8 cM and 22.8 cM, respectively (Fig 2), which were comparable with previous studies [18, 42]. The linkage analysis and genetic map confirmed that the pea powdery mildew resistance gene in G0001778 is an *er1* allele, *er1*-6 (Fig 2).

### 3.4 Development of a functional marker of *er1*-6

**Fig 3:**
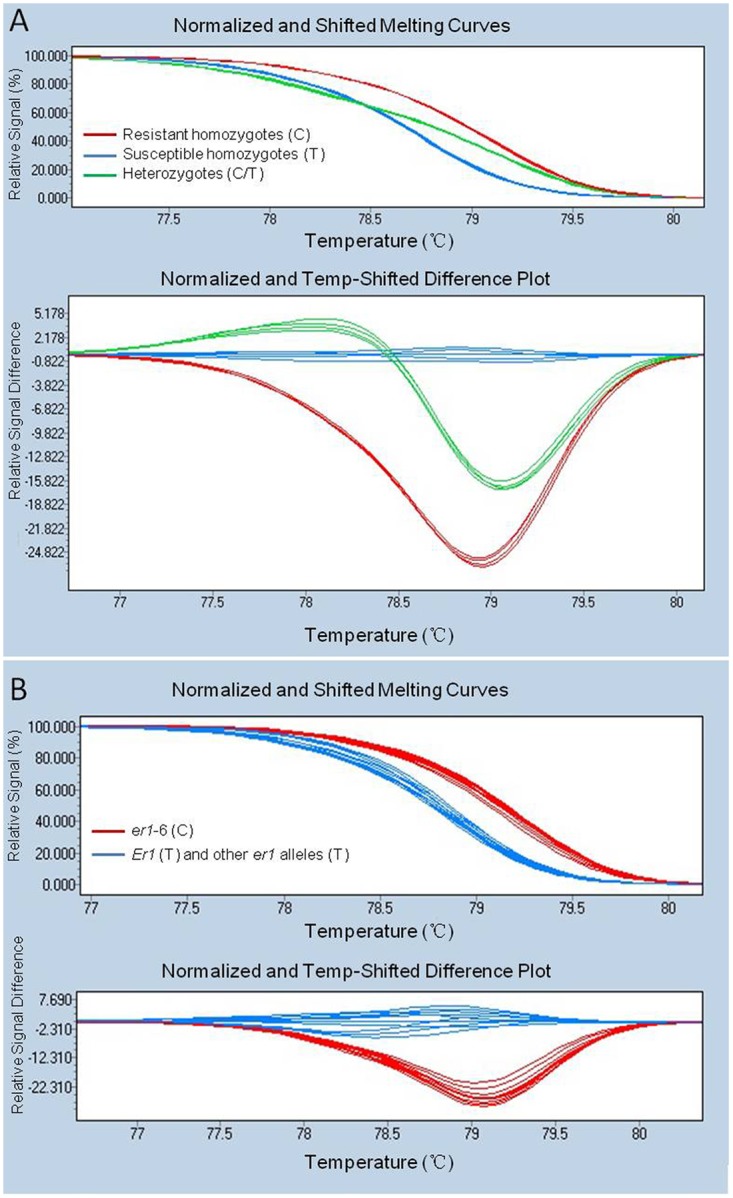
High-resolution melting (HRM) curve profiles of amplicons produced by the SNP marker SNP1121. A. HRM showing three distinct curves in F_2_ individuals derived from G0001778 (C) × Bawan 6 (T), corresponding resistant homozygotes, susceptible homozygotes and heterozygotes, respectively. B. HRM showing two different curve profiles in resistant pea landraces carrying *er1*-6 alleles (C), and both susceptible and resistant pea germplasms respectively carrying *Er1* (T) and other *er1* alleles (T).

Among the five primers flanking the mutation site (1121), only the PCR products amplified by primer pair *er1*-6-4F/4R (5′- CTGGAGATCACCTTTTCTGGTT -3′ / 5′- CATGTACAAACACACATACACACG -3′) showed a polymorphic melting curve between the contrasting parents G0001778 and Bawan 6 using the HRM assay. Primers *er1*-6-4F/4R amplified 158-bp fragments in both parents G0001778 and Bawan 6 (S3 Fig) and showed two distinct melting curves with a single melting peak, which was designated as SNP marker, SNP1121 (Fig 2). Subsequently, SNP1121 was used to analyze the genotypes of the whole F_2_ population using the HRM assay. Three distinct HRM melting curve profiles appeared, corresponding to resistant homozygotes, heterozygotes, and susceptible homozygotes of the F_2_ population from G0001778 × Bawan 6 (Fig 3A). Finally, to validate the effectiveness of the functional marker specific to *er1*-6, SNP1121 was used to distinguish resistant pea landraces carrying the *er1*-6 allele from other resistant pea germplasms carrying the other *er1* alleles (*er1*-1, *er1*-2, *er1*-4) and susceptible pea germplasms to *E*. *pisi* carrying the susceptible *Er1* gene, using the HRM assay. Two distinct HRM melting curve profiles appeared between the resistant landraces carrying *er1*-6 and the other resistant germplasms, Tara [40], X9002 and G0005576 [42], and YI [13] respectively carrying other *er1* alleles (*er1*-1, *er1*-2, *er1*-4) and the five susceptible pea germplasms (Bawan6, Lonwan 1, G1747, G3839, G3840) (Fig 3B).

## Discussion

Powdery mildew is an economically important disease of pea and significantly affects the quality and quantity of pea production [36, 37]. In China, research on resistance to pea powdery mildew is at an early stage. Recently, Wang et al. [39] indicated that resistant resources for pea powdery mildew were included in Chinese pea landraces, especially those from Yunnan province. The present study was carried out to screen for resistant landraces among 322 accessions from China using artificial inoculation under controlled greenhouse conditions, which helped us to avoid the ambiguities resulting from the influence of environmental factors in the field. The experimental design included not only susceptible cv. Bawan 6, but also resistant lines X9002 and YI, carrying the *er1* alleles [13, 42], which ensured the validity of the screening results.

We identified 33 resistant pea landraces from 11 Provinces or Autonomous Regions. No resistant landraces were found in the other seven provinces. This result is comparable with that of Wang et al. [39], who found resistant resource in landraces from Yunnan, but not from Shanxi, Shaanxi, Inner Mongolia and Qinghai, which indicated that the distribution of resistant resources may be associated with geographic origins.

Among the 33 landraces identified as resistant to *E*. *pisi*, 18 (54.5%) accessions were collected from Yunnan. Moreover, we found that 11 out of 12 immune landraces, three out of four highly resistant landraces and four out of 17 resistant landraces were collected from Yunnan. Yunnan locates at low latitude plateau region in China and has the complex landform, which results in its multiple climatic features and variable ecological environment [54]. Yunnan is an important centre of diversity for field pea in China due to its special geographical and climatic features [53, 55]. Zong et al. [53] revealed that pea germplasms from Yunnan showed high levels of genetic diversity. Thus, the variable climate in Yunnan could have resulted in different natural mutations in the *PsMLO1* gene for powdery mildew resistance compared with those in other geographic regions. Yunnan is a region which experiences extreme biotic and abiotic stresses [55, 56]. The presence of high frequency of resistant landraces harboring new allele *er1*-6 in Yunnan is considered as a result of specific adaptation to local climate and higher selection pressure of powdery mildew.

To date, only *er1* has been found to provide durable broad-spectrum resistance to *E*. *pisi* among the three known single inherited resistance genes (*er1*, *er2*, and *Er3*) [26, 27, 57]. Currently, *er1* resistance is the only one to be used widely for breeding purposes because of its complete and stable resistance [28]. All resistant commercial pea cultivars carry a recessive *er1* gene [12, 19, 21, 29]. Recently, Humphry et al. [13] revealed that the resistance conferred by *er1* is a result of loss-of-function mutations of *PsMLO1* [13, 34]. To date, five *er1* alleles have been reported in pea germplasms resistance to *E*. *pisi* [35]. Humphry et al. [13] identified four *er1* alleles in resistant pea germplasms, named *er1*-1 (JI1559), *er1*-2 (JI2302), *er1*-3 (JI210) and *er1*-4 (JI1951), representing naturally occurring mutations in *PsMLO1*. Simultaneously, Pavan et al. [34] identified another *er1* allele, *er1*-5, in pea line ROI3/02 during a mutagenesis program. Recently, Santo et al. [58] identified another two alleles, *er1mut1* and *er1mut2* caused by one single nucleotide mutation, in pea powdery mildew-resistant lines induced by chemical mutagenesis with ethylnitrosourea. Four of the five *er1* alleles are caused by point mutations in *PsMLO1*, including a base pair deletion or substitution, except for *er1*-2. Allele *er1*-2 was caused by a large DNA insertion of unknown size and identity in *PsMLO1*, which led to aberrant transcription [13, 34, 42].

In China, little is known about inheritance mode and resistance genes for powdery mildew in Chinese pea germplasms, except for pea line X9002 [42] and cv. Xucai 1 [59]. Zeng et al. [38] indicated that pea line X9002 was stably resistant to powdery mildew. Recently, Wang et al. [42] revealed that X9002 carries a recessive gene for powdery mildew resistance, which was mapped in a region containing the *er1* locus on pea LG VI, using molecular markers AD60 and c5DNAmet. Subsequent *PsMLO1* cDNA sequence analysis confirmed that resistance in X9002 was conferred by the *er1-*2 allele [42]. Recently, using the same analysis applied for X9002, Sun et al. [59] revealed that resistance in Chinese pea cv. Xucai 1 was also conferred by the *er1-*2 allele. Mutations identical to those in X9002 and Xucai 1 also occurred in previously described resistant pea cultivars and lines, such as Stratagem (JI 2302) [13], Franklin, Dorian, and Nadir [34]. The present study obtained cDNA sequences of *PsMLO1* at the *er1* locus from the identified 16 resistant pea landraces and controls. Based on 10 cloned sequences, the susceptible (Bawan 6) and resistant (X9002 and YI) controls produced identical *PsMLO1* cDNAs to their respectively previous studies, which confirmed the accuracy of cDNA sequencing of the target gene, *PsMLO1* [13, 42]. The resistant pea landrace G0005576 from Chongqing produced three distinct transcripts, characterized by a 129-bp deletion, and 155-bp and 220-bp insertions in *PsMLO1* (S2 Fig). All three transcripts were identical to resistant pea germplasms carrying the *er1*-2 allele [13, 42]. This indicated that resistance in G0005576 was also conferred by *er1*-2. Interestingly, the insertions of 155-bp and 220-bp are very similar (96% and 95%, respectively) to a sequence repeated five times in a pea genomic BAC clone sequence (GenBank accession number CU655882). The inserted 220-bp sequence is also highly similar (~87% identity) to part of a giant *Ogre* retrotransposon (GenBank, accession number AY299395) in the pea genome [58, 60].

The cDNA sequences of the *PsMLO1* gene in the other 15 resistant pea landraces showed identical transcripts, except for deletions or insertions of small fragments in one or two out of 10 clone sequences from a few accessions, which were caused by alternative splicing events. Compared with the wild-type *PsMLO1* cDNA sequence, all 15 resistant landraces showed a novel mutation site (position 1121) in *PsMLO1*, which was considered a new *er1* allele, named *er1*-6 (Fig 1). Interestingly, although the 15 resistance landraces carried the same resistance allele, *er1*-6, their phenotypic reaction to *E*. *pisi* showed slight differences in resistance level, from immunity to highly resistant with scores of 0 and 1, respectively (Table 2). These different phenotypes in the 15 resistance landraces indicate that expression of allele *er1*-6 may be influenced by interactions with related gene(s).

To study the inheritance of the resistant landrace G0001778 carrying the *er1*-6 allele, we obtained the phenotypic data of F_2_ and F_2:3_ populations derived from G0001778 × Bawan 6, which showed that resistance in G0001778 is conferred by a single recessive gene. Linkage analysis indicated that the polymorphic markers AD60 and c5DNAmet were linked to the resistance gene in G0001778 (Fig 2). The results of genetic mapping combined with the cDNA analysis of *PsMLO1* from G0001778 confirmed that resistance in G0001778 was conferred by a novel allele, *er1*-6 (Fig 2). To aid marker-assisted selection (MAS) of *er1*, Paven et al. [34] developed functional markers corresponding to the five known *er1* alleles. Highly informative cleaved amplified polymorphic sequence (CAPS) (*er1*-1 and *er1*-4), derived cleaved amplified polymorphic sequence (dCAPS) (*er1*-3), sequence tagged site (STS) (*er1*-2) and HRM (*er1*-5) markers were developed for the five *er1* alleles, respectively [34]. However, the functional markers were not verified in genetic populations. An STS marker for *er1*-2 was invalidated in our pea materials in a previous study [42]. Fortunately, another functional marker specific to *er1-*2, PsMLO1-650, a coupling-phase marker, was developed using the F_2_ population derived from Bawan 6 × X9002 [42].

HRM is a technique developed recently for scanning mutations, SNP detection and genotyping [52, 61, 62], and is used to determine the characteristic melting or disassociation behavior of PCR amplicons [63]. HRM markers are suitable for large-scale breeding programs based on high-throughput screening. Therefore, we developed successfully the functional marker SNP1121, which is associated with *er1*-6, based on a mutation in the *PsMLO1* gene, using the HRM technique. The forward and reverse primers of the SNP1121 marker were located in the 11th exon and 11th intron of the *PsMLO1* gene, respectively. The HRM assay using SNP1121 could successfully distinguish the contrasting parents, G0001778 and Bawan 6, and the marker SNP1121 co-segregated with phenotypes of F_2_ individuals derived from G0001778 × Bawan 6 (Fig 3A). Moreover, SNP1121 was validated and verified in other pea germplasms. HRM analysis using SNP1121 successfully distinguished resistant pea landraces carrying the *er1*-6 allele from resistant pea accessions carrying other *er1* alleles and germplasms susceptible to *E*. *pisi* (Fig 3B). We expect that SNP1121 will be effective in marker-assisted selection in pea breeding.

## Conclusions

This study identified 33 Chinese pea landraces resistant to *E*. *pisi* and discovered a novel resistance allele, *er1*-6, characterized by a point mutation (T→C) in position 1121 of the *PsMLO1* cDNA. Two markers, AD60 and c5DNAmet, were identified as linked to *er1*-6. A functional marker specific to *er1*-6, SNP1121, was developed successfully and verified in pea germplasms. Our results provided comprehensive genetic information for powdery mildew resistance and a powerful tool for marker-assisted selection in a pea breeding program.

## Supporting Information

S1 Fig Disease severity with a score of 0–4. (TIF)

S2 Fig Comparisons of cDNA sequence of *PsMLO1* gene from the wild-type pea cultivar Sprinter (Wild_type_PsMLO1) and the resistant pea landrace G0005576, based on 10 clones (G5576_cDNA1 to G5576_cDNA10). (TIF)

S3 Fig A single band of 158 bp was amplified using SNP marker SNP1121 in both parents, G0001778 and Bawan 6, and the derived F_2_ individuals. (TIF)

S1 Table Information for the five developed primer pairs flanking the mutation site (1121) associated with the *er1*-6 allele. (DOCX)
