# Supplementary figures and images for: Discovery of a Novel er1 Allele Conferring Powdery Mildew Resistance in Chinese Pea (Pisum sativum L.) Landraces
Source: PLoS One. 2016 Jan 25;11(1):e0147624. doi: 10.1371/journal.pone.0147624 (PMC4725671; doi:10.1371/journal.pone.0147624)

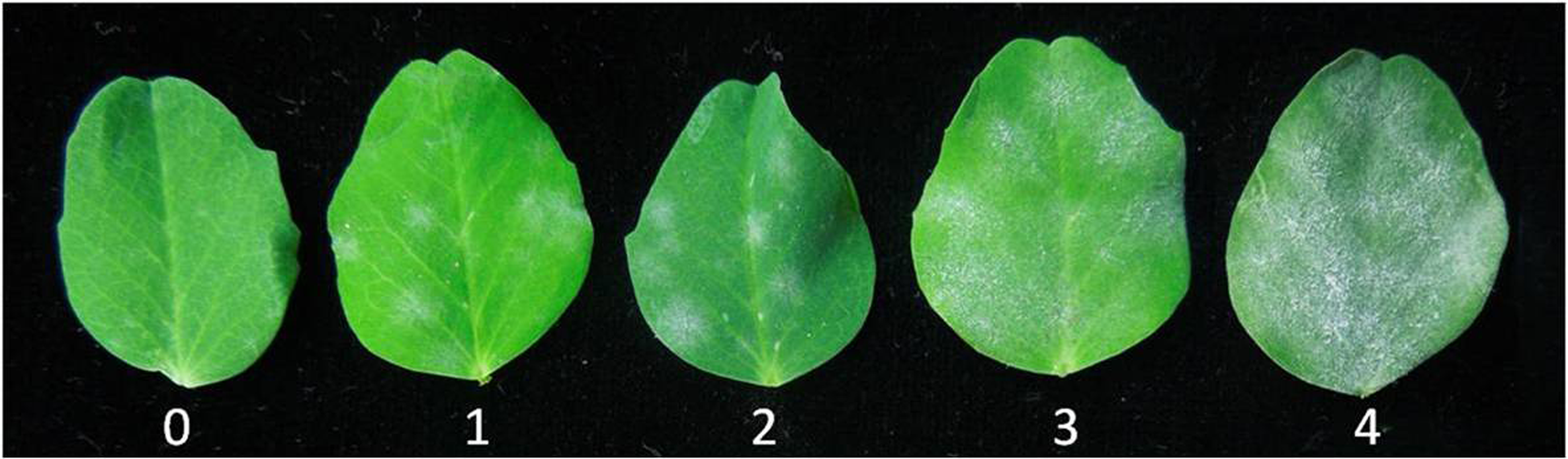

Supplement: S1 Fig — (TIF) [file pone.0147624.s001.tif]

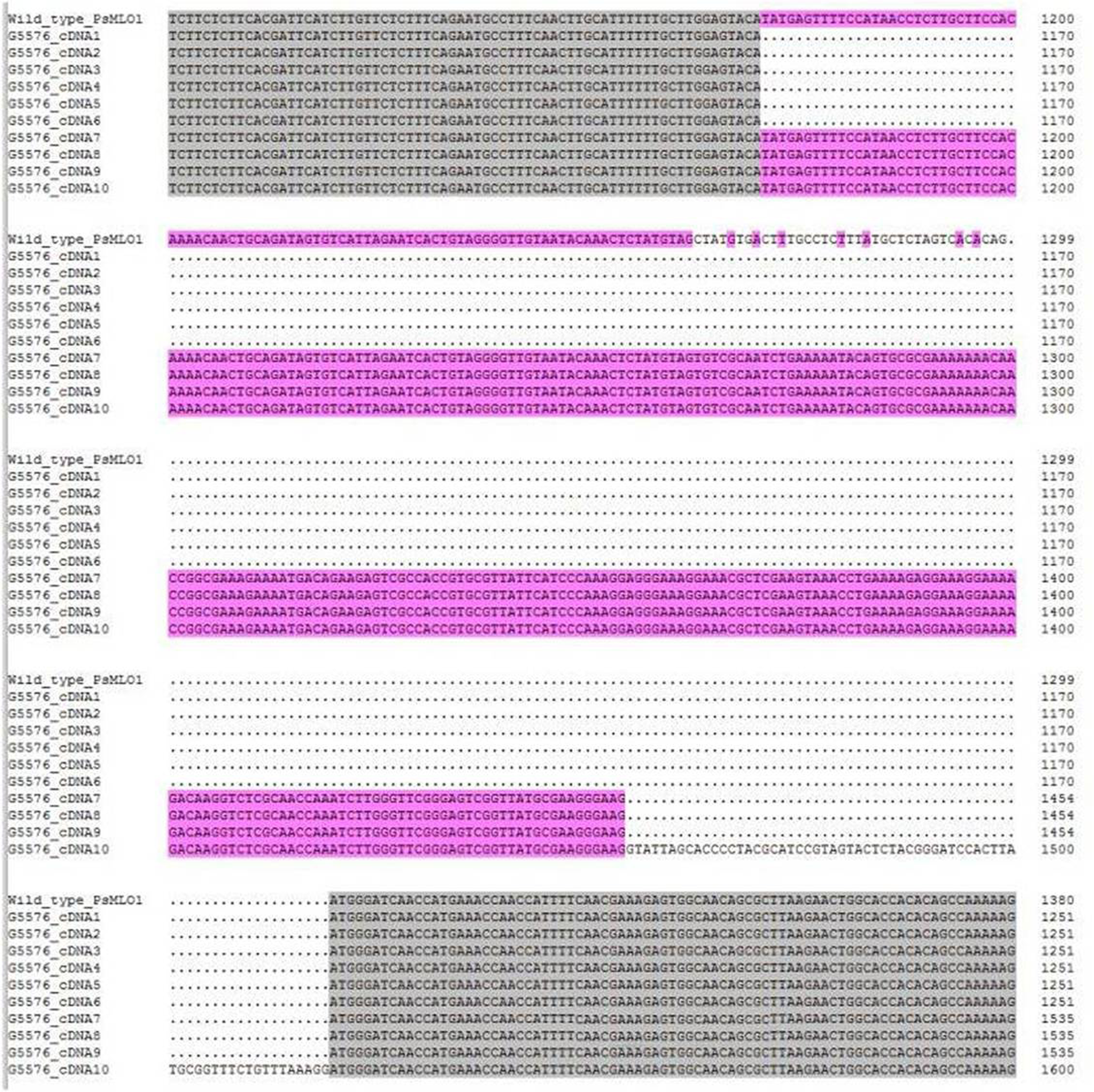

Supplement: S2 Fig — (TIF) [file pone.0147624.s002.tif]

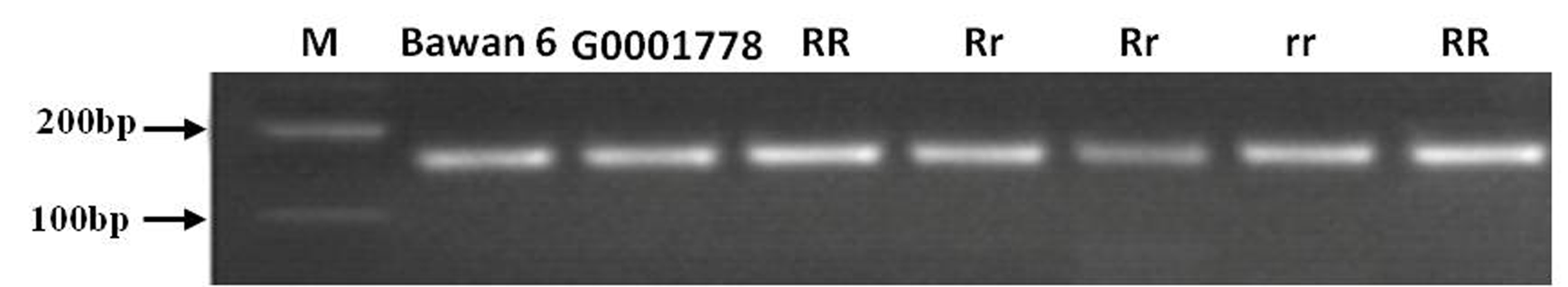

Supplement: S3 Fig — (TIF) [file pone.0147624.s003.tif]
