# Supplementary material for: Discovery of a Novel er1 Allele Conferring Powdery Mildew Resistance in Chinese Pea (Pisum sativum L.) Landraces
Source: PLoS One. 2016 Jan 25;11(1):e0147624. doi: 10.1371/journal.pone.0147624 (PMC4725671; doi:10.1371/journal.pone.0147624)
Supplement: S1 Table — (DOCX) [file pone.0147624.s004.docx]

**S1 Table.** **Information for the five developed primer pairs flanking the mutation site (1121) associated with the *er1*-6 allele.** (F: forward primer; R: reverse primer).

| Primer name | Primer sequence (5’—3’) | *T*m (°C) | PCR product (bp) |
| --- | --- | --- | --- |
| SNP1121-1F/R | F: TGGGATTAAGGATTCAAGACAG | 59 | 191 |
|  | R: CGCATGAGATTTGCATAGACA |  |  |
| SNP1121-2F/R | F: GGATTAAGGATTCAAGACAGAGGA | 60 | 189 |
|  | R: CGCATGAGATTTGCATAGACA |  |  |
| SNP1121-3F/R | F: ATCACCTTTTCTGGTTCAATCG | 59 | 152 |
|  | R: CATGTACAAACACACATACACACG |  |  |
| SNP1121-4F/R | F: CTGGAGATCACCTTTTCTGGTT | 59 | 158 |
|  | R: CATGTACAAACACACATACACACG |  |  |
| SNP1121-5F/R | F: TGGGATTAAGGATTCAAGACAG | 60 | 192 |
|  | R: GCGCATGAGATTTGCATAGAC |  |  |
